# RNF213 variant in a patient with Legius syndrome associated with moyamoya syndrome

**DOI:** 10.1002/mgg3.1669

**Published:** 2021-05-03

**Authors:** Giulia Romanisio, Cristina Chelleri, Marcello Scala, Gianluca Piccolo, Barbara Carlini, Laura Gatti, Valeria Capra, Federico Zara, Anna Bersano, Marco Pavanello, Patrizia De Marco, Maria Cristina Diana

**Affiliations:** ^1^ Pediatric Neurology and Neuromuscular Diseases Unit IRCCS Giannina Gaslini Institute Genoa Italy; ^2^ Department of Neurosciences, Rehabilitation, Ophthalmology, Genetics, Maternal and Child Health (DINOGMI) University of Genoa Genoa Italy; ^3^ Medical Genetics Unit IRCCS Giannina Gaslini Institute Genoa Italy; ^4^ Neurobiology Laboratory, Cerebrovascular Unit Fondazione IRCCS Istituto Neurologico Carlo Besta Milan Italy; ^5^ Cerebrovascular Unit Fondazione IRCCS Istituto Neurologico Carlo Besta Milan Italy; ^6^ Pediatric Neurosurgery Unit IRCCS Giannina Gaslini Institute Genoa Italy

Neurofibromatosis type 1 (NF1) is a clinically heterogeneous neurocutaneous genetic disorder that may occasionally be associated with various cerebrovascular anomalies, including moyamoya syndrome (Dlamini et al., 2019; Guey et al., 2015; Sam et al., 2015). A similar vascular involvement has been very recently reported in a patient with Legius syndrome, a rare condition characterized by café‐au‐lait spots and macrocephaly either with or without axillary and inguinal freckling, presenting with moyamoya syndrome (MMS) (Pabst et al., 2020). Although MMS has been largely reported in individuals with NF1, this recent association with Legius syndrome is relevant with regard to the phenotypic expansion of this rare condition. Legius syndrome has been long considered a milder clinical form of NF1, but it is a distinct autosomal dominant medical condition caused by pathogenic variants in *SPRED1* (OMIM *609291). We report the second case of moyamoya syndrome in a patient with Legius syndrome caused by a novel frameshift variant in *SPRED1*.

A 20‐year‐old Caucasian woman was clinically diagnosed with NF1 during the first years of life, according to the presence of multiple café‐au‐lait macules and axillary freckling. At the age of 4 years, she developed left‐arm hemiparesis and motor weakness. Brain magnetic resonance imaging (MRI) showed ischemic infarction of the right cerebellar hemisphere and whole‐body MRI revealed a mild aortic coarctation, without flow obstruction. At the age of 9 years, she presented with sudden generalized tonic–clonic seizures and brain MRI showed acute brainstem and left cerebellar hemisphere ischemia. One week later, an area of diffuse restriction in the left antero‐lateral portion of the pons‐midbrain junction was observed on brain magnetic resonance angiography (MRA) scans. No arterial narrowing could be noticed, but conventional angiography revealed tortuosity and irregularity of the distal branches of cerebellar arteries. One year later, MRA showed tortuosity and caliber irregularities of the superior cerebellar arteries, in combination with a moyamoya syndrome. Follow‐up brain MRAs and perfusion studies were stable. However, the electroencephalogram (EEG) at the age of 19 years showed bilateral anterior and posterior irritative abnormalities induced by hyperventilation, suggestive of the typical “Re‐build‐up” phenomenon observed in moyamoya disease. A prophylactic therapy with acetylsalicylic acid (ASA) was therefore started.

A targeted Next Generation Sequencing (NGS) panel including *NF1* (OMIM *613113), *SPRED1*, and *RNF213* (OMIM *613768) was performed within a retrospective study investigating the presence of *RNF213* pathogenic variants in Caucasian subjects with NF1/Legius syndrome and moyamoya syndrome. After written informed consent was collected, DNA was extracted from blood samples of the proband and the parents using QIAamp DNA Mini Kit according to the manufacturer's instructions (Qiagen S.A.). DNA concentrations were quantified using the Nanodrop ND‐1000 UV‐Vis spectrophotometer (Labtech France). We used the high‐throughput NGS machine Ion Torrent PGM in combination with Ion AmpliSeq Designer Software v4.2 (Life Technologies). After the identification of the candidate variant, the proband's peripheral blood was collected in PAXgene Blood RNA tubes and subjected to total RNA extraction using the PAXgene Blood RNA kit (Qiagen) for cDNA analysis (further data available in the Supplementary Material). NGS analysis led to the identification of the novel paternally inherited null variant NM_001256071.3:c.1471+1dupG; p.(?) in *RNF213* (NM_001256071.3, NP_001243000.2; https://www.ncbi.nlm.nih.gov/gene/57674) in our patient (Figure 1a) but, surprisingly, no pathogenic variant was observed in *NF1*. The patient carried instead the novel *de novo* NM_152594.3:c.330_331delGG; p.(Arg110Serfs*3) variant in *SPRED1* (NM_152594.3, NP_689807.1, https://www.ncbi.nlm.nih.gov/gene/161742) (Figure 1b), leading to a diagnosis of Legius syndrome. Both the *RNF213* and *SPRED1* variants are rare (absent from GnomAD database) and predicted damage by *in silico* tools. The sequencing of RT‐PCR products generated by the *RNF213* NM_001256071.3:c.1471+1dupG variant of the patient and wild‐type allele showed the breakpoint of junction between the first 82 bps of exon 8 and the first bases of exon 9 causing by the intronic variant, supporting the pathogenic role of this variant (Figure 1c,d).

**FIGURE 1:**
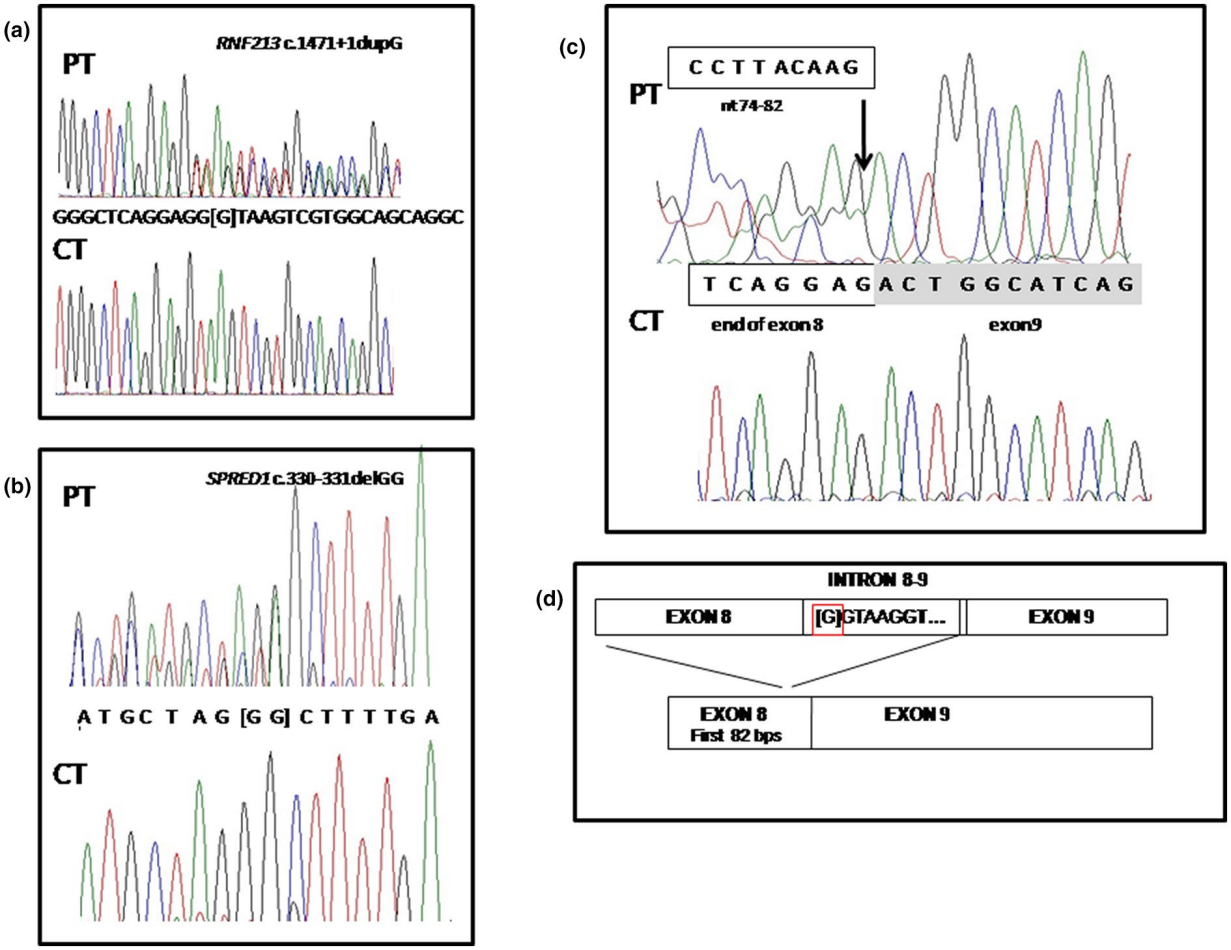
Genetic results. Sanger sequencing results and functional analysis of the RNF213 c.1471+1dupG. Electropherograms of the genomic sequence confirming the variants identified by NGS in the patient (PT), the *RNF213* c.1471+1dupG (a) and *SPRED1* c.330_331delGG (b). The wild‐type alleles of a control individual (CT) are also showed. (c) Partial sequence of RT‐PCR products generated by the *RNF213* c.1471+1dupG of the patient (PT) and by the wild‐type allele (CT) showing the breakpoint of junction between the first 82 bps of exon 8 and the first bases of exon 9 causing by the intronic variant. (d) Schematic representation of the alternative splicing of *RNF213* c.1471+1dupG variant. The position of intronic variant is highlighted by a red square

To our knowledge, this is the second patient with moyamoya syndrome and Legius syndrome to be reported in the literature. Of note, our patient was first diagnosed with NF1 based on her clinical features, but genetic testing revealed the correct diagnosis of Legius syndrome. Since NF1 and Legius syndrome share common cutaneous manifestations, namely café‐au‐lait spots and skinfold freckling, the differential diagnosis between these two conditions can be difficult. This is especially true in young patients, in whom the additional NF1‐related clinical features (e.g., neurofibromas and Lisch nodules) may still be absent. According to the current NIH criteria (Neurofibromatosis., 1988), skin features are particularly relevant for a diagnosis of NF1. Indeed, NF1 is diagnosed in subjects fulfilling ≥2 of the 7 NIH criteria and café‐au‐lait macules (≥6 macules >5 mm in prepubertal or >15 mm in postpubertal individuals) and skinfold freckling (axillary and/or inguinal) both represent distinct diagnostic criteria (Neurofibromatosis, 1988). However, due to the multisystemic involvement and the severity of the clinical manifestations associated with NF1, it is crucial to achieve a correct diagnosis (NF1 versus Legius syndrome) in a patient with suggestive cutaneous manifestations, with genetic testing playing a pivotal role in this regard.

Both NF1 and Legius syndrome belongs to the group of RASopathies or RAS/mitogen‐activated protein kinase (MAPK) syndromes. These severe conditions are caused by germline variants in genes of the Ras/MAPK pathway. The observation of moyamoya syndrome features in NF1 and other RASopathies suggest the existence of genetic factors underlying moyamoya pathogenesis, with the surgical management of the vascular disorder in these conditions being particularly challenging (Scala et al., 2018). Moyamoya syndrome is considered a polygenic disease and several susceptibility genes and genetic loci have been identified so far (Sam et al., 2015). Several variants in *ACTA2* (OMIM *102620), *TGFB1* (OMIM *190180), *PDGFRB* (OMIM *173410), and *MRVI1* (OMIM *604673) have been identified in Caucasian patients with both Moyamoya disease (MMD) and MMS (Dlamini et al., 2019; Guey et al., 2015). However, a locus on chromosome 17q25.3 within the *RNF213* locus was identified as the first major gene locus for dominant MMD. *RNF213* encodes the mysterin, an enzyme with a poorly understood biological role. It has been recently supposed that this protein might stabilize lipid droplets, suggesting a possible link between MMD pathogenesis and fat metabolism (Sugihara et al., 2019). Some *RNF213* variants have been postulated to increase the risk of MMS in East‐Asiatic populations (Morimoto et al., 2016; Phi et al., 2016), while no specific association was observed in Caucasian patients. We identified a novel *RNF213* variant localizing to the splicing site of the intron 8 and likely impairing the splicing process. Intriguingly, this is the first *RNF213* splicing variant associated with the development of moyamoya syndrome. In contrast with most previously reported MMS and MMD patients (Lin et al., 2011), the posterior cerebral arteries were affected in our patient (Phi et al., 2015; Scott et al., 2004). The mechanisms underlying MMS in NF1 are poorly understood, but a possible role might be played by the dysregulation of the RAS‐MAPK pathway. This is in line with the observation of MMS in other RASopathies (Neurofibromatosis, 1988). SPRED1 protein plays instead a relevant role in inhibiting endothelial cell migration and proliferation through an impairment in cell motility and Rho‐mediated actin reorganization (Jansen et al., 2013). These observations suggest a possible role of SPRED1 deficiency in the pathogenesis of the formation of the abnormal vascular networks observed in MMS.

This case illustrates that cerebral vasculopathy in RASopathies is probably underestimated among asymptomatic patients, but can actually lead to permanent neurological outcomes. In line with AHA/ASA guidelines, MRI/MRA screening (preferably during childhood) represents the gold standard for early diagnosis and follow‐up. The evidence of a novel *RNF213* variant in a patient with Legius syndrome associated with early‐onset bilateral MMS might suggest that *RNF213* variants act as negative prognostic factors. Although this speculation is intriguing, further confirmation is needed. Indeed, it was not possible to perform a brain‐MRA and investigate the presence of cerebral vasculopathy in the patient's healthy father, harboring the same *RNF213* variant identified in the proband.

The prevalence of cerebral vasculopathy in NF1 is approximately 5%, ranging from 2.5 to 6.4%, whereas epidemiological data remain elusive for other RASopathies, due to their rarity (Scala et al., 2018). However, brain MRI can play a pivotal role in the identification of brain abnormalities associated with these conditions (e.g., Chiari I malformation, hydrocephalus, optic pathway glioma) and the contribution of MRA can be extremely relevant for the early detection of cerebral vasculopathy, with a possible positive impact on the overall patient management (Scala et al., 2018). Accordingly, we suggest to perform MRI/MRA screening not only in patients with NF1 but also in subjects diagnosed with other RASopathies. Furthermore, genetic testing for *RNF213* variants is advisable in order to identify patients who might deserve a more careful and close follow‐up due to a more severe clinical course.

## CONFLICT OF INTEREST

There is no conflict of interest to declare.

## AUTHORS’ CONTRIBUTIONS

Conception of the study: Giulia Romanisio, Cristina Chelleri, Marcello Scala, Marco Pavanello, Patrizia De Marco. Sample collection and preparation: Giulia Romanisio, Gianluca Piccolo, Barbara Carlini, Laura Gatti, Valeria Capra, Anna Bersano, Patrizia De Marco. Planning and preparations of experiments: Federico Zara, Patrizia De Marco. Analysis of the data: Cristina Chelleri, Marcello Scala, Patrizia De Marco. Collection of clinical data: Giulia Romanisio, Cristina Chelleri, Gianluca Piccolo, Laura Gatti, Valeria Capra, Anna Bersano, Marco Pavanello, Maria Cristina Diana. Writing of manuscript: Giulia Romanisio, Cristina Chelleri, Marcello Scala, Patrizia De Marco. Revisions of the manuscript: All Authors. Funding for the study: Ricerca Finalizzata Ministeriale 2019 “Empowering the pathophysiology and prognosis of Moyamoya Arteriopathy”.

## Supporting information

Supplementary Material

## Data Availability

The data that supports the findings of this study are available in the supplementary material of this article.

## References

[mgg31669-bib-0001] Dlamini, N. , Muthusami, P. , & Amlie‐Lefond, C. (2019). Childhood moyamoya: Looking back to the future. Pediatric Neurology, 91, 11–19. 30424960 10.1016/j.pediatrneurol.2018.10.006

[mgg31669-bib-0002] Guey, S. , Tournier‐Lasserve, E. , Hervé, D. , & Kossorotoff, M. (2015). Moyamoya disease and syndromes: From genetics to clinical management. The Application of Clinical Genetics, 8, 49–68. 25733922 10.2147/TACG.S42772 PMC4337618

[mgg31669-bib-0003] Jansen, F. , Yang, X. , Hoelscher, M. , Cattelan, A. , Schmitz, T. , Proebsting, S. , & Werner, N. (2013). Endothelial microparticle‐mediated transfer of microRNA‐126 promotes vascular endothelial cell repair via SPRED1 and is abrogated in glucose‐damaged endothelial microparticles. Circulation, 128(18), 2026–2038. 24014835 10.1161/CIRCULATIONAHA.113.001720

[mgg31669-bib-0004] Lin, N. , Baird, L. , Koss, M. , Kopecky, K. E. , Gone, E. , Ullrich, N. J. , Scott, R. M. , & Smith, E. R. (2011). Discovery of asymptomatic moyamoya arteriopathy in pediatric syndromic populations: radiographic and clinical progression. Neurosurgical Focus, 31(6), E6. 10.3171/2011.10.FOCUS11228 22133171

[mgg31669-bib-0005] Morimoto, T. , Mineharu, Y. , Kobayashi, H. , Harada, K. H. , Funaki, T. , Takagi, Y. , Sakai, N. , Miyamoto, S. , & Koizumi, A. (2016). Significant association of the RNF213 p. R4810K polymorphism with quasi‐Moyamoya disease. Journal of Stroke and Cerebrovascular Diseases, 25(11), 2632–2636. 27476341 10.1016/j.jstrokecerebrovasdis.2016.07.004

[mgg31669-bib-0006] Neurofibromatosis . (1988). Conference statement. National Institutes of Health Consensus Development Conference. Archives of Neurology, 45(5), 575–578. 3128965

[mgg31669-bib-0007] Pabst, L. , Carroll, J. , Lo, W. , & Truxal, K. V. (2020). Moyamoya syndrome in a child with Legius syndrome: Introducing a cerebral vasculopathy to the SPRED1 phenotype? American Journal of Medical Genetics Part A, 185(1), 223–227. 33078527 10.1002/ajmg.a.61921

[mgg31669-bib-0008] Phi, J. H. , Choi, J. W. , Seong, M. W. , Kim, T. , Moon, Y. J. , Lee, J. , Koh, E. J. , Ryu, S. K. , Kang, T. H. , Bang, J. S. , Oh, C. W. , Park, S. S. , Lee, Z. Y. , Wang, K. C. , & Kim, S. K. (2016). Association between moyamoya syndrome and the RNF213 c.14576G>A variant in patients with neurofibromatosis type 1. Journal of Neurosurgery Pediatrics, 17, 717–722. 26849809 10.3171/2015.10.PEDS15537

[mgg31669-bib-0009] Phi, J. H. , Wang, K. C. , Lee, J. Y. , & Kim, S. K. (2015). Moyamoya syndrome: A window of Moyamoya disease. Journal of Korean Neurosurgical Society, 57(6), 408–414. 26180607 10.3340/jkns.2015.57.6.408 PMC4502236

[mgg31669-bib-0010] Sam, C. , Li, F. F. , & Liu, S. L. (2015). Inherited neurovascular diseases affecting cerebral blood vessels and smooth muscle. Metabolic Brain Disease, 30(5), 1105–1116. 25893882 10.1007/s11011-015-9668-y

[mgg31669-bib-0011] Scala, M. , Fiaschi, P. , Capra, V. , Garrè, M. L. , Tortora, D. , Ravegnani, M. , & Pavanello, M. (2018). When and why is surgical revascularization indicated for the treatment of moyamoya syndrome in patients with RASopathies? A systematic review of the literature and a single institute experience. Childs Nervous System, 34, 1311–1323. 10.1007/s00381-018-3833-7 29797062

[mgg31669-bib-0012] Scott, R. M. , Smith, J. L. , Robertson, R. L. , Madsen, J. R. , Soriano, S. G. , & Rockoff, M. A. (2004). Long‐term outcome in children with moyamoya syndrome after cranial revascularization by pial synangiosis. Journal of Neurosurgery: Pediatrics, 100(2), 142–149. 14758941 10.3171/ped.2004.100.2.0142

[mgg31669-bib-0013] Sugihara, M. , Morito, D. , Ainuki, S. , Hirano, Y. , Ogino, K. , Kitamura, A. , Hirata, H. , & Nagata, K. (2019). The AAA+ ATPase/ubiquitin ligase mysterin stabilizes cytoplasmic lipid droplets. Journal of Cell Biology, 218(3), 949–960. 10.1083/jcb.201712120 PMC6400562 30705059

