# Supplementary material for: RNF213 variant in a patient with Legius syndrome associated with moyamoya syndrome
Source: Mol Genet Genomic Med. 2021 May 3;9(6):e1669. doi: 10.1002/mgg3.1669 (PMC8222830; doi:10.1002/mgg3.1669)
Supplement: Supplementary file 1 — Supplementary Material [file MGG3-9-e1669-s001.docx]

**METHODS**

**NGS**

DNAs were extracted from blood samples using QIAamp DNA Mini Kit according to the manufacturer’s instructions (Qiagen S.A., Courtaboeuf, France). DNA concentrations were quantified using the Nanodrop ND-1000 UV-Vis spectrophotometer (Labtech France, Palaiseau, France). Written informed consent was obtained from the patients’ parents.

We used the high-throughput NGS machine, Ion Torrent PGM in combination with Ion AmpliSeq Designer Software v4.2 (Life Technologies). *NF1* (58 coding exons, 8661 bp) and *SPRED1* (7 exons, 7780 bp) amplicons were designed targeting the coding sequences of *NF1* and *SPRED1* genes and resulting in a total of 136 amplicons. The average target region with coverage ≥ 20X was 97.5% (NF1: 97.4%, SPRED1: 98.3%). In order to target the entire coding sequence of *RNF213* a second custom NGS panel was used composed by 244 amplicons showing the following features: 96.31% of total coverage, an amplicons range of 125 - 275 bp and an exon padding of 10 bp. The *RNF213* had a coverage of 95.9%. Amplicon library was prepared using the Ion AmpliSeq Library Kit 2.0: 2 multiplex primer pools were added to gDNA and amplified by PCR. The library was purified and quantified by Qbit 2.0 instrument; patients were pooled and loaded into an Ion 314 sequencing chip on Ion Torrent PMG machine and the runs were performed using Ion PGM 200 Sequencing kit with 500 flows.

The sequence reads were analyzed using the Ion Reporter pipeline and the CLC Genomics Workbanch 6.5.1 software (Qiagen), for alignment, variant detection and coverage analysis. Variant call was performed with a minimum coverage of 20 reads (≥ 20X). All of the called variants were validated by Sanger sequencing; their predicted functional effect was assessed by Polyphen 2.0 or by Mutation Taster software.

**RNA extraction, RT-PCR and RNF213 amplification.**

Peripheral blood was collected in PAXgene Blood RNA tubes and subjected to total RNA extraction using the PAXgene Blood RNA kit (Qiagen, Hilden, Germany) following the manufacturer instructions. RNA amount and quality were evaluated by NanoDrop (NanoDrop Technologies, Wilmington, DE, USA). Total mRNA extracted was retro-transcribed to cDNA using the SuperScript First-Strand Synthesis System kit (Invitrogen, Carlsbad, CA, USA) and then amplified with the Platinum PCR SuperMix High Fidelity kit (Invitrogen), using the primers encompassing exons 8-10 of RNF213 (NM_ 001256071): forward RNF213-8F (5’- gACCgCgTTCTTgTTgAAg**g**-3’) and reverse RNF213-10R (5’-gTgAAACTCATCTggTgAgC-3’) generating a wild-type PCR product of 694 bps.

**Sanger sequencing.**

PCR products were purified using the GenUP Exo SAP kit (BiotechRabbit, Berlin, Germany) and amplified by the Big Dye Terminator Cycle Sequencing kit (Applied Biosystems, Foster City, CA, USA).
